# Nutrtional and Phytochemical Characterstics of Fruits and Vegetable Wastes as Livestock Feed: A Case Study in Gamo Zone, Southern Ethiopia

**DOI:** 10.1155/2024/4427876

**Published:** 2024-10-30

**Authors:** Mitiku Yohannes, Yisehak Kechero, Yilkal Tadele

**Affiliations:** Department of Animal Sciences, Arba Minch University, P.O. Box 21, Arba Minch, Ethiopia

**Keywords:** antinutritional factors, food-feed competition, fruit and vegetable waste, macromineral, micromineral

## Abstract

Fruit and vegetable producers were creating a large amount of waste in homes, cafeterias, and agroprocessing units. The majority of this waste is composted and disposed in landfills and waterways. Recycling these wastes as animal feedstuffs will lessen food-feed competition and minimize environmental hazards. This study was carried out in the Gamo zone of southern Ethiopia, in the heart of the southern rift valley, to ascertain the nutritional profiles of fruit and vegetable by-products in relation to livestock feed potentials. All fruit and vegetable waste (FVW) samples were collected from homes, marketing facilities, restaurants, and waste disposal facilities. Proximate, detergent fiber component, minerals, and antinutritional factors were among the analyses performed on the collected samples. The results showed that, highest CP values recorded from banana leaf, 15.8 ± 0.8 (%DM) followed by avocado peel (14 ± 0.8) among fruit by-products and that of highest values of vegetable components were obtained from *Moringa oleifera* strip (20.6 ± 1.25) sweet potato (18.5 ± 1.55), respectively. The highest ME (MJ/kg DM) contents obtained from avocado and mango by-products among fruit wastes, while that of highest values recorded for cassava and potato peel (12.2 ± 0.4) and (11.3 ± 0.1) among vegetable components, respectively. The antinutrients included in FVW, namely, tannin, oxalate, and phytate, did not exceed the maximum permissible level for animal needs, which is less than 5% of DM. The macro- and micromineral profiles of these by-products also show encouraging results that contribute to maintaining the mineral needs of farm animals. FVW can be a viable and alternative source of supplemental feed for farm animals that primarily rely on low-quality natural pasture and crop residues, and can partially replace more expensive feedstuffs and their efficient reuse would minimize environmental impacts associated with the disposal of such wastes.

## 1. Introduction

The need for alternative livestock feed rises significantly if the consumption of animal products rises in the future. Because of the effects of climate change, competition for fuel and food, deforestation, water scarcity, loss of biodiversity, and other issues, meeting the demand for feed in a sustainable way will be challenging. The two obvious ways to be sustainable in crop-livestock mixed farming are efficient use of the feed resources already in place and expanding the pool of feed resources basis, with an emphasis that do not compete with human food [1]. Using these nonfood components from agricultural products as animal feed will lessen the environmental impact of their disposal [2, 3]. Theories and recommendations on nontraditional feed resources that can be utilized as feed for animal, along with some conservation techniques, feeding management of fruits and vegetable wastes and their by-products, have received much attention recently.

The need for and principles of sustainable livestock production apply universally with consideration of the multiple roles of livestock in economic growth, food security and poverty reduction. In addition, in pastoral and agropastoral areas, livestock is a key asset for poor communities, fulfilling multiple economic, social, and risk management functions. Moreover, livestock production contributes to food security directly by increasing producers' food diversity and availability, but also that of urban consumers and indirectly through income generation and increased farm resilience. Yet, the available feed resources are not fed in the right proportion as per the requirements of animals [3, 4]. Moreover, it has been challenged by many factories around the world. The climate changes mainly the long-term misbalance of temperature, wind, and rainfall characteristics of specific regions which has been exaggerated on the livestock productions [5].

Regardless of the huge numbers of livestock possessions in Ethiopia, the current contribution of livestock to the producers and to the national economy is by far lower than its size [6]. It has increasingly been unable to meet the demands of the rapidly growing population. Among the many factors that could contribute to the disproportionate role of the sector, it is inadequacy and poor quality of available feed resources for livestock [7, 8]. In mixed crop-livestock production systems, crop residue is the main feed source next to natural pasture [9]. Recently, linking waste management and sustainable animal feed production from agricultural by-products have gain substantial interest for both environmental issues and meeting the feed crisis of livestock population against the challenges imposed by various biophysical factors [10].

Farmed animals have a role in circular food systems: waste stream biomass can be used as feed, and farmed animals provide manure and pond sediment, which can be used as fertilizer to maintain or improve soil fertility [1]. Hence, efficient use of the available feed resources with minimum wastage and the widening of the feed resource base with novel feed ingredients, particularly those not competing with human food, will prove a buffer stock for farmers [10, 11]. The provision of new nontraditional alternative feed sources may be crucial in addressing this shortfall.

Based on the report of GZAD [12], and (personal observation), the recent agricultural production of the study area was characterized as cultivation of several tropical fruits like banana, mango, avocado, papaya, and vegetables like cabbage, tomato, potato, cassava, sweet potato, pumpkin, watermelon, and Enset, both on a small and large scale production [13].

In line with this, substantial amount of by-products generated from fruit and vegetable farms during harvesting and processing are primarily discarded as waste in open fields. An estimated amount of (more than 1.5 million metric tons per year), of fruit and vegetable by-products were discarded with the exception of emerging traditions of the local community providing as feed for their livestock in the area. There were no credible research findings or related information, mainly about fruit and vegetable-based by-products, their feed values, chemical and phytochemical analysis in Ethiopian settings. Therefore, the current study sought to exploit the nutritional characteristics of feed biomass samples from fruit and vegetable by-products, through determinations of proximate composition, detergent fiber fractions, mineral profiles, metabolizable energy, and antinutritional factor contents.

## 2. Materials and Methods

### 2.1. The Study Area

The study was conducted in the Gamo zone, Arba Minch University, in the southern rift valley of Ethiopia. The area is geographically located at 5° 57°–6° 71′N and 36° 37′–37° 98′E, and the general elevation of the Gamo zone ranges from 680 to 3568 m above sea level (m.a.s.l.). All sample biomasses collected from the area and chemical analysis were carried out in Arba Minch University, Hawassa University, and Addis Ababa University center of advanced nutrition and food science laboratories.

### 2.2. Fruit and Vegetable By-Product Sample Collection and Preparation

Banana by-products such as leaf, peel, and pseudostem biomass were collected from farms and marketing facilities. Fresh avocado and mango fruit seed kernels and peels were collected from farms, homes, hotels, and cafeteria services in the area. Vegetable and tuber crop by-products such as cabbage leaf striping, discarded tomatoes, *Moringa oleifera* stem, potato peel, sweet potato leaf and vein, and cassava peel and leaf were collected from residents, farms, restaurants, and hotel service providers in the area. All sample fruit and vegetable by-products were then physically prepared, separated from the hard seed coat/peel, and allowed to dry up under shade for 1 week. Fresh avocado and mango seed kernels were manually crushed with a large mortar and piston and partially dried for 2 days under shade, followed by oven drying at 55°C for 24 h at the laboratory. The complete sample of feed biomasses were prepared, grinded, and packaged into plastic containers, then brought to the laboratory for all chemical analysis. Three independent samples of each types of all by-products were prepared and labeled in triplicates with codes of which were used to have a clear statistical analysis.

### 2.3. Chemical and Metabolizable Energy Analysis of the Sample

The dry matter (DM), crude protein (CP) and ash contents of the feed samples determined according to [14]. The protein content was determined by using the Kjeldahl technique, which was detailed in AOAC [14], and calculated as *N* × 6.25. The acid detergent fiber (ADF) and acid detergent lignin (ADL) were assessed in accordance with Van Soest and Robertson [15]; whereas neutral detergent fiber (NDF) content was determined in accordance with Van Soest, Robertson, and Lewis [16]. Ether extract (EE) content was determined by using the Soxhlet device through Ankom apparatus and petroleum ether based on the procedures of [14]. Hemicellulose is estimated by subtracting %ADF from %NDF. The metabolizable energy (ME, MJ/kg) value was estimated from the adopted formula of Flynn, Schroeder, and Sakakibara [17] given below:

(1) $$\begin{array}{c} \text{ME}=\left(0.17\times \%\text{DDM}\right)-2.0. \end{array}$$

The most common and known anti-nutritional factors such as phytate, oxalate, and tannin were among determined components of the current study sample. The phytate concentration was determined by using UV_VIS spectrophotometer as indicated by Reddy and Love [18]. The oxalate concentration was determined by using [14] as indicated by Day and Underwood [19]. The tannin contents determined by using vanillin-HCL assay methods through a UV spectrophotometer as indicated in Van Buren and Robinson [20]. The macro and microminerals such as; iron, zinc, calcium, magnesium, potassium, and copper were analyzed by using an atomic absorption spectrophotometer. Phosphorus was determined by using vanado-molybdate reagent through colorimetric methods.

### 2.4. Statistical Analysis

One-way variance analysis was used to see mean differences among fruit types (banana by-products (leaf, pseudostem, and peel), mango, and avocado seed and peel). A similar procedure was employed for vegetable by-products to test mean differences among all vegetable by-products. These analyses were performed using GLM procedures of SAS version 9.3 statistical software following the statistical model *Y*_ij_ = *µ* + *P*_*j*_ + Σ_ij_, where *Y*_ij_: response variable *µ*: overall mean effect; *P*_*j*_: *i*^th^ effect of FVW types (banana leaf, peel pseudo stem, avocado peel/seed mango seed cabbage leaf, tomato fruit, *Moringa oleifera stem*, potato peel, sweet potato leaf and vein, cassava leaf and peel); and Σ_ij_ is the random error. Duncan's multiple range tests were used for mean separation. The mean differences were considered significant at (*p* ≤ 0.05). All laboratory analysis was done in duplicate.

## 3. Results

### 3.1. Nutritional Characteristics of Fruit By-Product Samples

The proximate and detergent fiber composition of thrown fruit by-products in the region, particularly different parts of banana, avocado, and mango is indicated in (Table 1). The proximate and fiber contents of banana leaf, peel mango seed, and avocado seed exhibited with statistical variation (*p* < 0.05) for all banana/avocado peel and banana pseudostem. The highest CP value was obtained from banana leaf, avocado peel and the lowest was from banana pseudostem. The ash contents also obtained from the samples indicate that avocado peel, banana leaf, and peel have higher values (*p* < 0.05) as compared to mango seed; avocado seed and banana pseudostem.

Ether extract (EE) contents obtained from the sample fruit wastes as indicated on table were observed with significant variation (*p* < 0.05). The highest values were recorded from avocado peel and seed, followed by banana leaf, mango seed, and banana pseudo stem; however, the lowest EE values were determined for banana peel.

The NDF contents of the fruit by-product samples varied significantly (*p* < 0.05). Mango seed and avocado seed have nearly comparable values, whereas banana fractions have higher ADF and NDF values (*p* < 0.05). ADL values recorded from the current sample showed significantly higher values obtained from banana parts, followed by mango seed, avocado seed, and avocado peel. Moreover, metabolizable energy values of the fruit waste sample showed with statistical variation (*p* < 0.05), having higher values obtained from mango and avocado by-products as compared to banana by-products in general.

### 3.2. Proximate and Detergent Fiber Content of Vegetable and Tuber Crop By-Products

The proximate and fiber contents of vegetable and tuber crop by-products are indicated in (Table 2). The dry matter content of the sample by-products varied significantly (*p* < 0.05), with higher values of *M. oleifera* strip, cassava leaf, sweet potato vein and potato peel, followed by tomato fruits, sweet potato leaf, cassava peel, and the lowest value recorded from cabbage leaf. The CP contents of all vegetable by-product samples used in the current study were exhibited with statistical variation (*p* < 0.05), having higher values obtained from *M. oleifera* strip and sweet potato leaf, followed by cabbage, tomato, sweet potato vein, cassava leaf fractions, and the lowest values obtained from potato and cassava peel, respectively. Similarly, the ether extract contents of the sample also indicated variation (*p* < 0.05) among vegetable by-products, with higher values obtained from sweet potato and cassava by-products, followed by *M. oleifera* strip and potato peel, while the lowest values were obtained from cabbage and discarded tomato fractions, respectively.

The fiber components, NDF, ADF, and ADL, of the current study sample showed statistical variation (*p* < 0.05), having higher values recorded for cassava parts, followed by sweet potato fractions, cabbage leaf, and tomato fruits, respectively. The ash contents also varied significantly (*p* < 0.05), with higher values for cabbage leaf, followed by *M. oleifera* strips and tomato fruits, respectively. The metabolizable energy values of vegetable by-products were significantly varied (*p* < 0.05), having higher values obtained from cassava peel and sweet potato leaf followed by cassava leaf and discarded tomato fruits, while the lowest values obtained from sweet potato vein, *Moringa oleifera* strips and potato peel, respectively.

### 3.3. Mineral Profiles of Fruit Waste Sample

The macro- and micromineral contents of fruit waste samples are indicated in (Table 3). Among fruit by-products, banana peel has a relatively higher total mineral content, followed by mango seed, avocado seed, and avocado peel, respectively.

The result indicates that other macrominerals such as P, Mg, and K values varied significantly (*p* < 0.05), with higher values recorded for banana peel, mango seed, and avocado seed and lower values obtained from avocado peel, banana pseudo stem and banana leaf, respectively.

Among microminerals, zinc and copper values were higher for avocado/mango seed and avocado peel, but lower values were recorded for banana parts. Iron contents also varied significantly (*p* < 0.05), with higher values obtained from banana peel and mango seed; however, similar lower values were recorded for avocado by-products, respectively.

### 3.4. Mineral Contents of Vegetable and Tuber CropBy-Products

The macro- and micromineral profiles of vegetable and tuber crop by-products are indicated in (Table 4). Results indicated statistical variation (*p* < 0.05) for all macrominerals, with higher values of *M*. *oleifera* followed by cabbage leaf and similar values of cassava leaf, tomato, potato peel, and sweet potato by-products for calcium and potassium, respectively.

Similarly, higher values of phosphorus content were recorded from sweet potato fraction and potato peel, similar values were obtained from all other vegetable by-products. Magnesium and potassium values indicated higher values for *M. oleifera* strips, followed by tomato, cassava leaf, cassava peel, and cabbage leaf, but lower values obtained from potato peel. Among microminerals, higher values were obtained from *M. oleifera* strips for both iron and zinc, and higher values of copper were obtained from cassava leaf as compared to other vegetable by-products.

### 3.5. Antinutritional Factors of the Sample Fruit Wastes

Major antinutritional factors of the fruit by-product samples are indicated in (Table 5). As indicated in the table, statistically varied (*p* < 0.05) for all anti-nutritional factors and a relatively higher concentration of tannin was recorded for banana by-products, followed by mango/avocado seed and avocado peel, respectively.

The concentration of oxalate was higher for banana leaf/pseudo stem and avocado peel, followed by avocado seed and banana peel. Similarly, the phytate concentration of the sample indicated as higher for avocado peel and banana by-products except banana pseudostem as compared to other by-products.

### 3.6. Antinutritional Factors of the Sample Vegetable and Tuber Crop Wastes

Vegetable and tuber crop by-products for anti-nutritional factor concentration are indicated in (Table 6). The contents of tannin, oxalate, and phytate for all sample by-products significantly varied (*p* < 0.05). The concentration of tannin is higher for *M. oleifera* strips and potato peel, followed by cassava and sweet potato leaf, but lower for tomato and cassava peel, respectively.

The oxalate content also indicated higher values for cassava peel, followed by cassava and cabbage leaf, but lower values were recorded for sweet potato by-products, *M. oleifera* strips, and tomato fruit, respectively. Moreover, a relatively higher content of phytate was recorded from cassava by-products, followed by cabbage leaf and potato peel, but lower values were recorded from *M. oleifera* strips and sweet potato by-products, respectively.

## 4. Discussions

### 4.1. Chemical Composition of Fruit By-Products

The proximate composition and fiber components of all fruit by-products were observed with statistical variation, mainly due to plant species variation, soli type, and types of cultivars in the area. Among proximate analyses, nearly comparable values of DM, lower CP, and fiber components of banana fractions were recorded as compared to the study findings of Chali et al. [21]. The CP contents investigated from the current sample of banana peel are slightly higher than (5.13 ± 0.14) and (7.3%) reported by Redondo-Gómez et al. [22] and Abou-Arab and Abu-Salem [23], respectively. The CP value of avocado seed is slightly lower than Tugiyanti, Iriyanti, and Apriyanto [24], Arukwe et al. [25]; and Ejiofor et al. [26], (15.55 ± 0.36), (17.94 ± 1.40), and (15.58 ± 0.18), respectively, and higher than that of 1.61 ± 0.87% and 5.77% reported by Gladys [27] and Tugiyanti, Iriyanti, and Apriyanto [24], respectively. Moreover, the CP content of mango seed is in accordance with (10.06 ± 0.12) reported by Fowomola [28] and higher than 5.90% and 3.244% of mango seed reported by both Okoruwa and Omoragbon [29] and Orayaga [30]. The study finding of Sahoo et al. [10] shows that FVWs are rich in many phytochemicals, vitamins, energy, and minerals but slightly have low protein contents. However, the value of CP obtained from this study shows that fruit wastes except banana pseudostem have a medium level, which might be similar and superior to the majority of grains and can play a great role in promoting the growth of farm animals.

Except banana pseudostem, all other fruit by-products can satisfy the minimum maintenance requirements of farm animals, as indicated by NRC [31]. The difference observed might be attributable to species variation, agroecological conditions, and anatomical structure of plants and the natural physico-chemical characteristics of plant varieties. The ash content of banana leaf in the current study is lower than the 15.5% reported by [21]. However, it was higher than the results (2.71%, 2.26 ± 0.23) from avocado seed powder reported by Tugiyanti, Iriyanti, and Apriyanto [24], and Ejiofor et al. [26], respectively. Generally, the ash content of fruit wastes represents total mineral contents and is directly linked with soil mineral contents, the application of different fertilizers, and the up taking capability of the plants in the area.

### 4.2. Chemical Composition of Vegetable and Tuber Crop By-Products

The proximate and fiber components of determined vegetable and tuber crop by-products were observed with significant variation (*p* < 0.05). Among the proximate composition of the current study samples, the discarded cabbage leaf had lower DM content, but it was in the ranges of the values reported by [32] and Nkosi et al. [33], of four species of brassica vegetables and specific *Brassica oleracea*, respectively. However, except dry matter all other proximate and fiber content value of cabbage leaf sample in current study was lower than the reported values of [34, 35], respectively. All proximate and fiber analysis values of the discarded tomato sample in the current study are lower than the findings of Redondo-Gómez et al. [22] and Ventura, Pieltain, and Castanon [36], for the DM, CP, NDF, and ADF and are nearly in similar ranges for ADL and Ash contents, respectively. The DM and fiber contents of potato peel obtained from the current sample are higher than (47%–52%), but the CP and ash were in accordance with the ranges of (8%–16%) and (4.5%–5.5%), respectively, reported by Martha [37]. The chemical composition of *M. oleifera* strips and sweet potato by-products (leaf and vein) was observed to have higher feed quality ranges for DM, CP, EE, NDF, ADF, and ADL contents, but the ash contents lie in a similar range as compared to other vegetable by-products.

The CP values of cassava peel and leaf were lower than (43.5 ± 2.3) and higher than (4.5/%DM) (4.4%) reported by Oresegun et al. [38], and Idugboe, Nwokoro, and Imasuen [39], respectively. Cassava leaf and peel exhibited higher EE and NDF values, nearly comparable values of ADF, ADL, and ash as compared to all other vegetable by-products. The differences in all proximate composition, fiber components, and ash contents might be attributed to the variation in genetic makeup and mode of preparation, soil types, and agroecological conditions. For example, as the study finding by Redondo-Gómez et al. [22], indicates they have used the extracted tomato pomace from the processing industry as feed ingredient in their findings, while fresh discarded tomato used as sample in the current study and other several edaphic factors could be considered as influential factor in general.

### 4.3. Mineral Contents of Fruit Wastes

The determined minerals of fruit by-products varied significantly (*p* < 0.05), primarily due to the variation of types of biomasses collected in the area. Banana by-products such as leaves, peels, and pseudostems were rich in both macro- and microminerals. However, banana peel had higher levels of all macro- and microminerals except zinc and copper, followed by leaf and pseudostem, respectively. The mineral contents of banana peels obtained from the current sample are lower than those of reported by Oyeyinka and Afolayan [40], in calcium, potassium, phosphorus, and magnesium but higher in zinc, iron, and copper contents. Similarly, both macro- and micromineral contents of banana by-products obtained from the current sample were lower than those of Afzal et al. [41], and Abou-Arab and Abu-Salem [23], as both authors declared that genotype variation and mode of preparation like microwave drying methods can affect the concentration of minerals in banana cultivars and by-products in general. Moreover, the mango seed sample is higher (*p* < 0.05) in potassium, magnesium, copper, and iron contents but lies in a similar range for zinc, calcium, and phosphorus contents as compared to other fruit by-products. Similarly, avocado seed and peel also have higher values (*p* < 0.05) of magnesium, potassium, zinc, and copper as compared to other fruit by-products. Calcium and zinc contents of mango seed were slightly higher than (111.3 mg/100 g) and (1.10 mg/100 g) reported by Rai, Dash, and Behera [42], but lower results were recorded for iron and nearly similar for zinc contents.

Calcium and iron contents of avocado seed were slightly higher than (14.15 ± 3.01 mg/100 g) and (0.09 ± 0.01 mg/100 g), respectively, reported by Arukwe et al. [25]. Magnesium, phosphorus, and potassium contents of current results for avocado seed were lower than (26.16 ± 5.90 mg/100 g), (31.33 ± 6.11 mg/100 g), and (100.83 ± 5.64 mg/100 g) and nearly similar results of iron (0.31 ± 0.03 mg/100 g) were obtained from similar authors. Based on the predominant values of minerals in the current sample fruit wastes (banana by-products, mango seed, avocado seed, and peel), have important mineral ingredients to satisfy the demand of livestock, some of which are deficient in the majority of conventional feed resources available in the area. This implies that these by-products could provide important health benefits and may have a big impact on nutrient metabolism, electrolyte balance, and nutrient absorption. As a result, all components of fruit by-products have rich in both macro- and micromineral content that may meet the minimal mineral needs of farm animals.

### 4.4. Mineral Contents of Vegetable and Tuber Crop Wastes

Among macrominerals, calcium was higher in *Moringa oleifera* strips and cabbage leaf, but the current study results were lower than the findings of Masitlha, Eyassu, and Demel [43], and Anunciacao et al. [44], for *M. oleifera* leaf and cabbage leaf, and nearly in line with similar authors for phosphorus, potassium, magnesium, and micromineral contents, respectively. The iron contents of sweet potato by-products in the current sample were nearly in line with (0.53 and 0.73 mg/100 g) on a dry weight basis and slightly lower for all other elements, as reported by Sanoussi et al. [45], respectively. The mineral contents of cassava peel and leaf obtained from the current study sample were slightly higher than (0.30, 0.32, 0.57, and 0.01/%DM) for calcium, phosphorus, potassium and magnesium values, respectively, reported by Idugboe, Nwokoro, and Imasuen [39]. However, all the mineral contents of cassava leaf in the current study were lower than the findings of three Nigerian genotypes and different agroecological condition studies reported by Alamu et al. [46], from eastern Ethiopia. Similarly, the mineral profiles of vegetable and tuber by-products have optimum level of minerals, might be better than conventional feed resources and can fulfill the mineral requirements of farm animals.

### 4.5. Antinutritional Factors of Fruit By-Products

The antinutritional factors in banana by-products have been reported by several authors. Among the many antinutrients found in fruits, three important antinutrients, such as tannin, oxalates, and phytate, were determined in this particular study. Based on the report of Abou-Arab and Abu-Salem [23], banana by-products were rich in phytate, alkaloids, oxalate, hydrogen cyanides, and tannin. The current study finding of antinutritional factors found in banana by-products is relatively higher as compared to other fruit wastes but lower than the reported results of Abou-Arab and Abu-Salem [23], for banana cultivars in Egypt. Similarly, the values of avocado seed and peel were slightly higher in phytate and oxalate contents as compared to other fruit wastes but lower than the (12.87 ± 0.02 mg/100 g) for avocado seed reported by Talabi et al. [47], and Gladys [27], and comparable with the (3.18 ± 0.16 mg/100 g) of avocado seed reported by [26]. The oxalate content of avocado seed and peel is slightly lower than (4.07 ± 0.01 mg/100 g) and (3.92 ± 0.86 mg/100 g) reported by Talabi et al. [47], and Gladys [27]. However, the value of oxalate from mango seed is lower than that of (1.49 ± 0.01 mg/100 g), but nearly comparable values of tannin were reported by Fowomola [28]. The variation of current results and others could be related to soil, climatic conditions, agroecologies, genetic variation of fruits, and mode of preparation, as microwave heat treatment could potentially affect the chemical and phytochemical contents of fruit by-products [23]. Generally, low levels of anti-nutrients were recorded from all available fruit by-products which might be considered less toxic and did not adversely affect the health status and production of farm animals.

### 4.6. Antinutritional Factors of Vegetable and Tuber Crop Wastes

Various concentrations of tannin, oxalate, and phytate in available vegetable and tuber by-products were determined in order to test their effects on nutritional components and physiological functions of farm animals. Among the determined by-products, cassava peel and leaf had relatively higher concentrations of phytate and oxalate as compared to other by-products, followed by potato peel and sweet potato by-products, but lower than the average values of (1.4% and 1.2% DM) contents of phytate and tannin for cassava leaf reported by Oresegun et al. [38], respectively. Similarly, the phytate content value of cassava peel in the current study is lower than the mean phytic acid concentration of cassava tuber reported by Addisu and Negussie [48] in eastern Ethiopia. Generally, the oxalate concentration can tend to bind with calcium in food, thereby rendering calcium inaccessible for the ordinary physiology of farm animals. The tannin concentration may bind with protein and fiber, which could affect accessibility and reduce microbial digestion of feed particles, and the phytate concentration found in plant materials is known for its chelating impact on certain essential mineral elements, for instance, Ca, Mg, Fe, and Zn, to form insoluble phytate salts that can affect the health of farm animals, as pinpointed by Addisu and Negussie [48]. However, all anti-nutrient contents (tannin, oxalate, and phytate) determined in the current study are considered to be within safe limits.

## 5. Conclusion

The study revealed that all fruit, vegetable, and tuber by-products have valuable nutritional compounds capable of maintaining the nutrient requirements of farm animals in the area. The findings indicated statistical variation among proximate composition, fiber components, mineral profiles, and antinutritional factors of all fruit by-products (banana by-products, mango by-products, and avocado by-products). Relatively, higher CP values were obtained from banana leaf, avocado seed, and peel, followed by mango seed and banana peel, among fruit by-products. Similarly, higher values of CP were obtained from *M. oleifera* leaf strips, sweet potato by-products, followed by cabbage leaf and tomato among vegetable and tuber wastes. Among minerals, banana peels represented a good source of all macronutrients and iron, followed by avocado and mango by-products. Avocado and mango by-products are good sources of microminerals. The antinutrient contents of all fruit, vegetable, and tuber by-product samples were present at reasonable levels (< 5% of DM) and were harmless to farm animals. Generally, all components of fruit and vegetable by-products have a great dietary role and possibly contribute to animal rations. Therefore, dietary incorporation of a certain amount of these discarded fruit and vegetable by-products in the ration of ruminants could be considered a functional feed ingredient, supplementary source, useful in substitution of commercial concentrate, could be considered a useful alternative feed resource, together with other conventional feed type, can maintain and sustain the minimum nutrient requirement of farm animals in an area where dry season feed shortage drastically affect production and productivity of farm animals.

## Figures and Tables

**Table 1:** Proximate and detergent fiber (% DM basis) and metabolizable energy (kcal/kg DM) contents of fruit wastes.

Fruit wastes	DM	CP	EE	NDF	ADF	ADL	Ash	HC	ME
Banana leaf	49.5 ± 2.3^a^	15.8 ± 0.8^a^	4.8 ± 0.6^a^	42.8 ± 5.2^a^	28.7 ± 2.4^a^	10.3 ± 1.3^a^	6.7 ± 0.6^a^	14.3 ± 3.1^b^	8.9 ± 0.1^b^
Banana peel	45.4 ± 0.7^b^	8.4 ± 1.2^c^	2.7 ± 0.2^c^	32.6 ± 3.3^c^	27.3 ± 1.7^a^	9.8 ± 2.4^a^	8.5 ± 0.5^a^	6 ± 1.4^c^	9.5 ± 0.6^b^
Banana pseudostem	46.7 ± 1.5^b^	4.7 ± 0.3^d^	3.5 ± 0.7^c^	46.7 ± 2.5^a^	24.2 ± 1.1^b^	7.8 ± 1.8^b^	4.7 ± 0.7^c^	22.7 ± 0.2^a^	7.8 ± 0.5^b^
Avocado seed	48.7 ± 1.5^a^	12.6 ± 0.5^b^	4.5 ± 0.3^a^	31.3 ± 1.6^b^	24.8 ± 1.0^b^	3.3 ± 0.3^c^	4.2 ± 0.4^b^	6.3 ± 0.4^c^	10.2 ± 1.1^a^
Avocado peel	41.6 ± 0.6^b^	14.8 ± 0.4^a^	5.6 ± 0.2^a^	17.8 ± 1.2^d^	10.1 ± 0.6^d^	2.6 ± 0.4^c^	8.3 ± 0.4^a^	9 ± 0.1^c^	11.8 ± 1.4^a^
Mango seed	49.4 ± 0.7^a^	10.7 ± 0.6^b^	3.8 ± 0.3^c^	35.4 ± 1.5^b^	20.8 ± 0.8^c^	4.4 ± 0.2^c^	5.8 ± 0.6^b^	14.6 ± 0.4^b^	10.4 ± 0.8^a^
*p* values	< 0.031	< 0.020	< 0.002	< 0.03	< 0.001	< 0.001	< 0.001	< 0.001	< 0.03

Abbreviations: ADF, acid detergent fiber; ADL, acid detergent lignin; CP, crude protein; DM, dry matter; EE, ether extract; HC, hemicellulose; ME, metabolizable energy (MJ/kg DM); NDF, neutral detergent fiber.

^a,b,c,d^mean with different superscripts down to the column shows significant variation (*p* < 0.05).

**Table 2:** Proximate and detergent fiber (% DM basis) and metabolizable energy (MJ/kg DM) contents of vegetable and tuber crop wastes.

Vegetable wastes	DM	CP	EE	NDF	ADF	ADL	Ash	HC	ME
Cabbage (*Brassica ol.*)	37.4 ± 1.7^c^	17.3 ± 0.5^b^	4.6 ± 0.7^c^	25.4 ± 0.7^c^	16.5 ± 1.2^c^	4.4 ± 0.2^a^	8.7 ± 0.4^a^	7.8 ± 0.9^c^	11.0 ± 0.2^b^
Discarded tomato	46.5 ± 2.3^b^	17.2 ± 2.2^b^	2.5 ± 0.7^c^	26.4 ± 1.4^c^	12.6 ± 0.8^c^	2.3 ± 0.2^b^	5.3 ± 0.3^b^	13.7 ± 1.6^a^	10.6 ± 0.3^b^
Potato peel	49.4 ± 1.7^a^	8.4 ± 0.9^c^	5.7 ± 0.8^b^	27.3 ± 1.6^c^	18.5 ± 1.2^b^	6.2 ± 0.4^a^	4.4 ± 0.2^c^	8.7 ± 1.34^c^	8.8 ± 0.6^c^
Sweet potato leaf	46.7 ± 1.6^b^	18.5 ± 1.5^a^	10.7 ± 0.6^a^	30.4 ± 0.8^b^	16.3 ± 1.2^c^	4.6 ± 0.7^a^	3.7 ± 0.5^c^	14.1 ± 0.8^a^	11.3 ± 0.1^a^
Sweet potato vein	49.6 ± 1.4^a^	15.6 ± 0.8^b^	13.8 ± 0.2^a^	35.3 ± 1.2^a^	20.7 ± 0.4^a^	6.8 ± 0.3^a^	4.6 ± 0.4^c^	14.7 ± 1.2^a^	9.5 ± 0.6^c^
*Moringa oleifera* strip	49.8 ± 1.8^a^	20.6 ± 1.2^a^	6.4 ± 1.8^b^	36.5 ± 1.5^a^	24.4 ± 1.5^a^	4.4 ± 0.3^a^	5.6 ± 0.3^b^	12.1 ± 1.4^b^	9.2 ± 0.4^c^
Cassava peel	44.8 ± 1.3^b^	8.4 ± 0.4^c^	10.4 ± 0.8^a^	32.3 ± 1.3^a^	17.6 ± 1.1^b^	4.8 ± 0.5^a^	2.3 ± 0.4^c^	14.6 ± 1.4^a^	12.2 ± 0.4^a^
Cassava leaf	49.7 ± 0.8^a^	13.6 ± 0.8^b^	10.6 ± 0.3^a^	28.7 ± 1.7^b^	19.1 ± 0.8^b^	3.5 ± 0.3^b^	3.4 ± 0.6^c^	9.5 ± 1.9^c^	10.1 ± 0.2^b^
*p* values	0.013	< 0.001	< 0.001	< 0.001	0.021	< 0.001	< 0.001	< 0.002	< 0.032

Abbreviations: ADF, acid detergent fiber; ADL, acid detergent lignin; CP, crude protein; DM, dry matter; EE, ether extract; HC, hemicellulose; ME, metabolizable energy (MJ/kg DM); NDF, neutral detergent fiber.

^a,b,c,d^mean with different superscripts down to column shows significant variation (*p* < 0.05).

**Table 3:** Least square means for mineral contents of fruit by-products.

Fruit type	Macrominerals (mg/g)				Microminerals (mg/100 gm)		
	Ca	P	Mg	K	Fe	Zn	Cu
Banana leaf	1.6 ± 0.8^b^	0.6 ± 0.1^b^	0.8 ± 0.04^b^	2.5 ± 1.03^b^	2.8 ± 0.07^b^	0.3 ± 0.0^c^	0.04 ± 0.05^c^
Banana peel	3.4 ± 1.3^a^	2.4 ± 0.6^a^	2.3 ± 0.6^a^	6.8 ± 0.7^a^	3.6 ± 0.4^a^	0.7 ± 0.01^b^	0.05 ± 0.0^c^
B. Pseudostem	0.4 ± 0.02^c^	1.6 ± 0.7^b^	0.4 ± 0.04^c^	0.6 ± 0.03^c^	1.04 ± 0.01^b^	0.9 ± 0.01^a^	1.3 ± 0.6^b^
Mango seed	1.4 ± 3.1^b^	1.8 ± 0.6^b^	1.2 ± 2.6^a^	4.5 ± 0.6^a^	6.2 ± 0.07^a^	0.5 ± 0.02^b^	6.5 ± 0.2^a^
Avocado seed	1.1 ± 0.7^b^	2.4 ± 1.1^a^	2.4 ± 1.2^a^	2.3 ± 0.5^b^	0.3 ± 0.07^c^	0.9 ± 0.01^a^	2.5 ± 0.3^b^
Avocado peel	0.3 ± 0.4^c^	2.7 ± 1.3^a^	0.8 ± 0.6^b^	0.7 ± 0.2^c^	0.07 ± 0.01^c^	1.02 ± 0.01^a^	4.2 ± 0.4^a^
*p* values	< 0.001	< 0.001	< 0.001	< 0.001	< 0.001	< 0.001	< 0.001

^a,b,c^mean differences down to the column shows significant variation (*p* < 0.05).

**Table 4:** Least square means for mineral contents of vegetable and tuber crop wastes.

Vegetable wastes	Macrominerals (mg/g)				Microminerals (mg/100 gm)		
	Ca	P	Mg	K	Fe	Zn	Cu
Cabbage leaf	2.8 ± 0.7^a^	0.5 ± 0.01^b^	0.8 ± 0.1^a^	0.7 ± 0.01^b^	1.2 ± 0.3^b^	0.6 ± 0.10^c^	1.3 ± 0.02^b^
Tomato	1.6 ± 0.02^b^	0.7 ± 0.00^b^	1.2 ± 0.5^a^	2.3 ± 0.02^a^	0.8 ± 0.01^c^	0.3 ± 0.01^c^	0.5 ± 0.0^c^
Potato peel	1.0 ± 0.04^b^	0.8 ± 0.02^a^	0.03 ± 0.0^d^	0.2 ± 0.01^c^	0.3 ± 0.0^c^	0.2 ± 0.0^c^	0.4 ± 0.0^c^
S. Potato leaf	1.4 ± 0.6^b^	1.2 ± 0.3^a^	0.7 ± 0.02^b^	0.6 ± 0.0^b^	0.5 ± 0.01^c^	0.3 ± 0.0^c^	0.6 ± 0.01^c^
S. Potato vein	1.1 ± 0.5^b^	0.9 ± 0.02^a^	0.4 ± 0.01^c^	0.7 ± 0.03^b^	0.4 ± 0.02^c^	0.04 ± 0.0^d^	0.04 ± 0.0^d^
*Moringa oleifera* strip	3.5 ± 1.1^a^	0.6 ± 0.0^b^	1.2 ± 0.7^a^	0.9 ± 0.01^a^	3.5 ± 0.7^a^	7.4 ± 0.4^a^	0.8 ± 0.2^c^
Cassava peel	0.7 ± 0.8^c^	0.6 ± 0.01^b^	0.2 ± 0.01^c^	1.0 ± 0.3^a^	0.6 ± 0.04^c^	1.5 ± 0.3^b^	0.7 ± 0.2^c^
Cassava leaf	1.7 ± 0.3^b^	0.7 ± 0.01^b^	0.8 ± 0.02^a^	1.1 ± 0.0^a^	0.8 ± 0.02^c^	2.5 ± 0.6^b^	2.5 ± 0.3^a^
*p* values	0.0001	0.0001	0.001	0.001	0.0001	0.0001	0.0001

Abbreviations: S = sweet, M = *Moringa*.

^a,b,c,d^mean with different superscripts down to the column shows significant variation (*p* < 0.05).

**Table 5:** Least square means for antinutritional factors of fruit wastes (mg/100 gm).

Fruit wastes/by-products	Tannin	Oxalate	Phytate
Banana leaf	6.4 ± 2.5^a^	4.3 ± 1.4^a^	6.3 ± 1.2^a^
Banana peel	3.6 ± 0.2^b^	1.05 ± 0.2^b^	5.8 ± 0.6^a^
Banana pseudostem	4.3 ± 0.6^a^	5.4 ± 0.8^a^	0.3 ± 0.01^c^
Mango seed kernel	2.04 ± 0.03^c^	0.5 ± 0.06^c^	1.8 ± 0.2^b^
Avocado seed kernel	2.6 ± 0.2^c^	1.08 ± 0.3^b^	1.8 ± 0.1^b^
Avocado peel	1.7 ± 0.2^d^	3.3 ± 0.3^a^	8.09 ± 0.5^a^
*p* values	< 0.001	< 0.001	< 0.001

^a,b,c^mean with different superscripts down to the column shows significant variation (*p* < 0.05).

**Table 6:** Least square means of antinutrients for vegetable and tuber crop by-products (mg/100 gm).

Vegetable wastes/by-products	Tannin	Oxalate	Phytate
Cabbage leaf	1.3 ± 0.04^c^	1.6 ± 0.02^b^	5.5 ± 0.01^b^
Discarded tomato	0.7 ± 0.03^d^	0.9 ± 0.01^c^	1.1 ± 0.02^cd^
Potato peel	3.3 ± 0.5^a^	1.3 ± 0.07^b^	5.3 ± 1.2^b^
Sweet potato leaf	2.3 ± 0.6^b^	0.6 ± 0.01^c^	2.3 ± 0.6^c^
Sweet potato vein	1.8 ± 0.7^c^	0.8 ± 0.02^c^	0.8 ± 0.05^d^
*Moringa oleifera* strip	3.4 ± 0.2^a^	0.2 ± 0.01^c^	1.02 ± 0.04^cd^
Cassava peel	0.8 ± 0.03^d^	2.8 ± 0.7^a^	6.6 ± 0.8^a^
Cassava leaf	2.5 ± 0.3^b^	1.8 ± 0.1^b^	8.3 ± 1.7^a^
*p* values	< 0.0001	< 0.0001	< 0.0001

^a,b,c,d^mean with different superscripts down to column shows significant variation (*p* < 0.05).

## Data Availability

Data will be made available on reasonable requests.
